# Mitochondrial Phylogenomics of Fagales Provides Insights Into Plant Mitogenome Mosaic Evolution

**DOI:** 10.3389/fpls.2021.762195

**Published:** 2021-10-18

**Authors:** Yanlei Feng, Xiaoguo Xiang, Delara Akhter, Ronghui Pan, Zhixi Fu, Xiaohua Jin

**Affiliations:** ^1^Institute of Biology, Westlake Institute for Advanced Study, Hangzhou, China; ^2^College of Agriculture and Biotechnology, Institute of Crop Science, Zhejiang University, Hangzhou, China; ^3^School of Life Sciences, Westlake University, Hangzhou, China; ^4^School of Life Sciences, Nanchang University, Nanchang, China; ^5^Department of Genetics and Plant Breeding, Sylhet Agricultural University, Sylhet, Bangladesh; ^6^College of Life Science, Sichuan Normal University, Chengdu, China; ^7^Sustainable Development Research Center of Resources and Environment of Western Sichuan, Sichuan Normal University, Chengdu, China; ^8^Institute of Botany, The Chinese Academy of Sciences, Beijing, China

**Keywords:** mitochondrial genome, Fagales, horizontal transfer, evolution, genome size variation

## Abstract

Fagales are an order of woody plants and comprise more than 1,100 species, most of which produce economically important timbers, nuts, and fruits. Their nuclear and plastid genomes are well-sequenced and provided valuable resources to study their phylogeny, breeding, resistance, etc. However, little is known about the mitochondrial genomes (mitogenomes), which hinder a full understanding of their genome evolution. In this study, we assembled complete mitogenomes of 23 species, covering five of the seven families of Fagales. These mitogenomes had similar gene sets but varied 2.4 times in size. The mitochondrial genes were highly conserved, and their capacity in phylogeny was challenging. The mitogenomic structure was extremely dynamic, and synteny among species was poor. Further analyses of the Fagales mitogenomes revealed extremely mosaic characteristics, with horizontal transfer (HGT)-like sequences from almost all seed plant taxa and even mitoviruses. The largest mitogenome, *Carpinus cordata*, did not have large amounts of specific sequences but instead contained a high proportion of sequences homologous to other Fagales. Independent and unequal transfers of third-party DNA, including nuclear genome and other resources, may partially account for the HGT-like fragments and unbalanced size expansions observed in Fagales mitogenomes. Supporting this, a mitochondrial plasmid-like of nuclear origin was found in *Carpinus*. Overall, we deciphered the last genetic materials of Fagales, and our large-scale analyses provide new insights into plant mitogenome evolution and size variation.

## Introduction

The order Fagales of flowering plants belong to the Rosids clade of the Eudicotidae. Fagales contain more than 1,100 species in seven families and 33 genera, according to the Angiosperm Phylogeny Group (APG) system (Sennikov et al., 2016). They are one of the most critical woody plants that grow in tropical, subtropical, and temperate forests (Xiang et al., 2014). Many Fagales play significant roles in ecosystem support and food supply, including beeches, oaks, birches, and some nuts and fruits, such as walnuts, chestnuts, hazels, and bayberries. Some species can fix nitrogen *via* root nodules through symbiosis with bacteria.

Fagales is one of the most sequenced orders in angiosperms. To date, the nuclear genomes of at least 28 species from five families have been sequenced (https://www.plabipd.de). Besides, >150 Fagales plastomes have been released. These genomes provide valuable genetic resources for improving nut quality and disease resistance, and these genomes also increased our knowledge of their phylogeny, nitrogen fixation, and sex determination (e.g., Griesmann et al., 2018; Jia et al., 2019; Lovell et al., 2021; Lucas et al., 2021; Yang et al., 2021). However, despite this, the last genome in the cell, the mitochondrial genome (mitogenome), is seldom studied in Fagales. So far, only three mitogenomes have been released, namely, *Betula pendula, Quercus variabilis*, and *Fagus sylvatica*. The *B. pendula* mitogenome was derived from whole-genome sequencing (WGS) study, but only minimal information regarding the mitogenome was included (Salojärvi et al., 2017). The *Q. variabilis* mitogenome was similarly sparsely described (Bi et al., 2019). *F. sylvatica* mitogenome was published recently (Mader et al., 2020). The evolution of mitogenomes in Fagales remains unanswered. Parsing the last unknown genetic material is crucial for understanding the evolution and genomic resources of Fagales.

Mitogenome in plants exhibits many unique features compared with those in animals and fungi. In angiosperms, its size is highly expanded and also varies significantly among species, ranging from 200 Kb up to 11 Mb (Sloan et al., 2012; exception see Skippington et al., 2015). Duplications and foreign DNA, including plastid-derived insertions (referred to as mitochondrial plastid insertions, MTPTs), nuclear insertions, and even horizontal gene/DNA transfers (HGTs), contribute significantly to the expansion (Mower et al., 2012; Wynn and Christensen, 2018). Plant mitochondrial DNA has the lowest substitution in the cell, while the structure is highly dynamic, with even close relatives or individuals of the same species exhibiting differences (Wolfe et al., 1987; Palmer and Herbon, 1988). Rearrangements between repeats could generate substoichiometric isomers, and mitogenomic chromosomes often exhibited unusual structures, e.g., multipartite or branched (Cheng et al., 2017; Gualberto and Newton, 2017; Kozik et al., 2019). These unique characteristics of plant mitogenomes hinder the production of complete and high-quality assemblies. In many plant species, the mitogenome has become the last genome that remains to be deciphered. Mitogenomic publications, to date, have usually focused on one or a few species, and large-scale comparisons are still scarce. The full scope of mitogenome evolution remains obscure.

In this study, we assembled complete mitogenomes of 23 Fagales species, including 16 genera from five families, covering almost half of the total Fagales genera and 71% of the total families, respectively. We showed that the mitogenomes in Fagales are extremely mosaic and rich in HGT-like sequences. Mitogenome size varies significantly among species and is likely affected by third-party DNA such as nuclear genome or some viruses. This is one of the few studies that comprise the largest number of new and complete angiosperm mitogenomes yet produced. It gives many comprehensive insights into the mitogenomic evolution in Fagales as well as in angiosperms.

## Materials and Methods

### Sequenced Data Acquisition

Raw reads used for our assembly were all obtained from the NCBI SRA database (https://www.ncbi.nlm.nih.gov/sra). Fagales genome sequencing projects were retrieved from SRA, and finally, 23 species from 16 genera and five families were selected to perform the assembly (Supplementary Table 1). All the data were whole-genome sequencing, meaning that reads included sequences from the nuclear, mitochondrial, and plastid genomes. Organelle genome sequences are usually smaller than nuclear sequences but are present at much higher copy numbers. Therefore, relatively small amounts of data were enough to obtain mitogenomes and plastomes in Fagales.

### Genome Assembly

Raw reads of each species were filtered for low-quality bases using TRIMMOMATIC v0.36 (Bolger et al., 2014). Clean reads of ~2–4 Gb were used for *de novo* assembly with SPADES v3.13 (Bankevich et al., 2012) (Supplementary Table 1). Plastid contigs of *Casuarina equisetifolia, Lithocarpus fenestratus*, and *Quercus suber* were obtained by BLASTN v2.9.0 (Camacho et al., 2009) searches of all assembled contigs against the *B. pendula* plastid genome (plastome, GenBank ID: NC_044852). Clean reads were then mapped to plastid contigs using GENEIOUS R10 (Biomatters, Inc.), and contigs were extended and connected manually until joined. Inverted repeat (IR) boundaries were identified by searching repeats using the GENEIOUS “Repeat Finder” plugin. Mitogenomes are often more variable than plastomes in terms of DNA sequences and structure. Preliminary mitogenome contigs were identified from total contigs by BLASTN with the *B. pendula* mitogenome (GenBank ID: LT855379) as a reference (word size: 16, evalue: 1e-20). All hit sequences longer than 500 bp were extracted. Two subsequent strategies were used to improve completeness and sequence content. For completeness, contigs were annotated using the GENEIOUS “Annotate from Database” function, where the “Database” comprised all known mitochondrial genes. If known mitochondrial genes were absent, reads were mapped to the genes (of reference) to check coverage and confirm the presence or absence. Mitochondrial genes that were missing from preliminary contigs were used to search all contigs, and identified contigs were added to the preliminary mitogenome contig set. This strategy ensured that gene sets for mitogenome assemblies were complete. For DNA content, clean reads were mapped back to the selected contigs. Plastid (higher coverage) and other contigs (unbalanced coverage) were removed from the set to provide approximate mitogenome coverage, which was then used to bait other potential mitochondrial contigs from all contigs. Newly selected contigs were mapped back by reads, and nonmitochondrial contigs were removed as before. This strategy reduced the amount of missing sequences and ensured that mitogenome assemblies were as complete as possible.

Next, the comprehensive mitochondrial contig sets were joined together. Contigs normally ended with repeat and/or MTPT sequences. Repeats longer than 50 bp in contigs were found using GENEIOUS “Repeat Finder,” and paired reads were mapped to contigs. Repeat regions were identified and resolved using sequencing coverage. Connections of long repeats may introduce artificial rearrangements. MTPTs are very similar to plastome sequences, and it is not usually possible to assemble MTPTs directly into contigs. MTPTs can be identified on plastomes (or plastid contigs); however, unlike repeat sequences, MTPT regions cannot be easily resolved by coverage as plastome coverage is usually much higher than mitogenome coverage. Repeats were filled and contigs were connected at both ends, after which MTPT ends were mapped to the plastome. After plastome mapping, the closest ends in the same orientation were most likely derived from the same MTPT. Rearrangements or recombination can occur within MTPTs, resulting in extended distances between sequences or opposite orientations with respect to plastome mapping. In these circumstances, paired reads could be used to identify the correct connections. MTPTs and their plastid counterparts may not be 100% identical, and additional steps were needed to correct MTPTs identified in the previous step. Reads were re-mapped and the divergent bases were manually checked and corrected, and reads that were 100% identical to the plastome were filtered, with the remaining “unused reads” re-mapped to mitogenomes to enhance the identification of divergent bases.

Several iterations of the map-check-connect strategies outlined above were usually sufficient to resolve all the repetitive and MTPT ends and retrieve one or more circular chromosomes. As the last step, paired-end reads were re-mapped a final time to check and correct any misassemblies and ensure that all bases were correct. The processes of the assembly are depicted in Supplementary Figure 1.

### Annotation

Putative mitochondrial protein-coding and rRNA genes were annotated by similarity to known mitochondrial genes, followed by manual corrections, and tRNA genes were predicted using tRNAscan-SE v2.0 (Chan and Lowe, 2019). Coding genes with disrupted reading frames, premature stop codons, or non-triplet frameshifts were annotated as pseudogenes.

Mitochondrial plastid insertions were determined by BLASTN comparison to a collection of plastomes. Hits smaller than 100 bp were masked. Dispersed repeats within the genome were searched by BLASTN against itself. Hits with identity <95% were filtered. Repeat lengths were determined using a custom Perl script. Only one part of each repeat pair was calculated, and overlapping bases were counted only once.

### Phylogeny

Four datasets were prepared for the phylogenetic reconstructions: (1) 43 mitochondrial genes, including introns and three rRNA genes; (2) 40 mitochondrial protein-coding sequences (CDSs), in which RNA-edited sites were predicted using the PREP website (Mower, 2009) and removed manually (an edited site within a codon prompted the removal of corresponding codons in all species); (3) 78 plastid CDSs; (4) nuclear 45S sequences (18S, 5.8S, and 25S rRNAs and the spacer regions), which were obtained from *de novo* contigs. Sequences were aligned by MAFFT software with “auto” mode (Katoh and Standley, 2013) and then concatenated into one matrix. Maximum-likelihood (ML) trees were built using IQTREE v1.6.12 with parameter “*-bb 1000 -m 476 GTR*+*G4*+*F -me 0.0001 runs 10*” (Nguyen et al., 2015). The used accessions are shown in Supplementary Table 3.

### Genus-Specific Sequence (GSS) Analysis

A BLAST program was used to compare mitogenomes to a database comprising all Fagales mitogenomes, with an e-value of 1e-5 and word size of 16. GSSs i.e., sequences present only within the specific Fagales genus) longer than 300 bp were isolated using a custom Python script. Short hits short than 70 bp were masked. *Quercus* species exhibited non-monophyletic relationships (Figure 3), and *Q. robur* was considered as a single genus in the analysis. GSSs were searched against the NCBI *nt* database, with parameters as before, and each saved the first 100 hits. The best hits for each GSS were examined (more than one best hit was possible if sequences matched different targets) using a custom Python script. Only best hits longer than 100 bp were used, and MTPTs were removed from the results. Subsequently, the best matches were grouped into orders, and a face-to-face tree was plotted in R using the APE package cophyloplot function (Paradis et al., 2004). Connections were colored using RColorBrewer (https://colorbrewer2.org/), and orders were positioned with reference to the Angiosperm Phylogeny Group website (https://www.mobot.org/MOBOT/research/APweb/).

### Synteny Inference

Mitogenome syntenies between families were plotted using CIRCOS v0.69 (Krzywinski et al., 2009). Links were searched by BLASTN with default parameters and hits shorter than 500 bp were excluded. Syntenies within each family were plotted by Python version MCscan of JCVI utility libraries v1.1.17 (Tang et al., 2008). The mitogenomes were cut into 300-bp pieces and forced to use as genes to search orthologous regions with parameter –*cscore* = *0.99*. Syntenies between *Carpinus, Fagus* and, *J. microcarpa*, and other mitogenomes, respectively, were also plotted by MCscan with –*cscore* = *0.7*.

## Results

### Assembly and Completeness Assessment

Our assembly approach focused on solving disconnections caused by repeats and MTPTs, which are two main difficulties of mitogenome assembly. Sequencing coverage was used to resolve repeats; MTPTs were identified using their positions and directions on the plastome (Supplementary Figure 1). For each species, 2–3 Gb bases were used for the assembly, and the final coverage depth ranged from 33 to 174 (Supplementary Table 1). One disadvantage of short reads is their inability to process long repeats. The structure of our assemblies could only represent one potential type. Of the 23 species, 13 yielded one or more circular mitogenomes, and the remaining 10 species contained one or more linear chromosomes (Table 1). The mitogenome of *Fagus sylvatica* was previously assembled using both long and short reads to produce a single circular chromosome of 504,715 bp in length (Mader et al., 2020). The sequence content of the published assembly was almost identical to that of the *Fagus* assembly produced in this study, differing only in two bases. The only disparity between the two assemblies was an inversion of a sequence located between 900-bp repeats. The consistency between our assembly and that of the previous study provided support for the practicability and reliability of our assembly methods.

**Table 1:** Basic information of Fagales mitogenomes. In the column “Chr,” the number means the total number of chromosomes, while “C” and “L” behind represent “circular” and “linear,” respectively.

**Species**	**Family**	**length (bp)**	**Chr**	**GC (%)**	**CDS**	**tRNA**	**rRNA**	**Repeat (bp)**	**MTPT (bp)**
*Alnus glutinosa*	Betulaceae	629,389	16L	45.44	35	18	3	8,581	29,856
Betula pendula*	Betulaceae	581,505	1L	45.52	36	19	3	3,703	31,908
*Betula platyphylla*	Betulaceae	581,519	2L	45.53	36	19	3	3,724	32,032
*Carpinus cordata*	Betulaceae	922,154	3C	44.97	34	20	3	16,557	46,637
*Corylus avellana*	Betulaceae	635,030	2L	44.58	35	22	3	39,128	60,416
*Ostrya chinensis*	Betulaceae	688,786	1L	45.23	34	18	3	2,601	31,075
*Ostryopsis nobilis*	Betulaceae	669,332	1C	45.21	35	18	3	4,810	24,710
*Casuarina equisetifolia*	Casuarinaceae	492,230	2C	44.15	35	23	3	2,431	66,400
*Casuarina glauca*	Casuarinaceae	445,851	2C	44.92	34	19	3	1,758	21,776
*Fagus sylvatica*	Fagaceae	504,715	1C	45.85	34	17	3	2,702	4,615
*Castanea mollissima*	Fagaceae	388,038	1C	45.67	36	20	3	10,888	10,169
*Lithocarpus fenestratus*	Fagaceae	485,396	6L	45.76	35	17	3	13,254	7,122
*Quercus robur*	Fagaceae	390,878	1C	45.92	35	17	3	22,006	8,140
*Quercus suber*	Fagaceae	478,989	1L	45.85	36	19	3	26,635	4,967
Quercus variabilis*	Fagaceae	412,886	1C	45.76	36	17	3	20,747	4,537
*Cyclocarya paliurus*	Juglandaceae	628,759	1C	44.89	37	19	3	2,422	34,143
*Juglans cathayensis*	Juglandaceae	740,307	1C	45.16	37	20	3	25,019	22,632
*Juglans hindsii*	Juglandaceae	716,397	1C	45.25	36	17	3	2,118	12,514
*Juglans microcarpa*	Juglandaceae	623,287	1L	45.2	35	18	3	6,984	12,448
*Juglans nigra*	Juglandaceae	716,680	1C	45.26	37	17	3	2,022	12,667
*Juglans regia*	Juglandaceae	775,914	3L	45.19	35	17	3	16,232	15,813
*Juglans sigillata*	Juglandaceae	778,034	1L	44.87	36	17	3	19,731	34,425
*Platycarya strobilacea*	Juglandaceae	502,903	1C	45.26	37	19	3	3,253	28,548
*Pterocarya stenoptera*	Juglandaceae	603,233	1L	45.35	36	17	3	5,269	3,524
*Morella rubra*	Myricaceae	523,452	2C	45.33	38	18	3	5,668	9,312

*The two species with asterisks mean published by others. For Fagus, we used our assembly*.

### Mitogenome Size and Content

**Figure 1:**
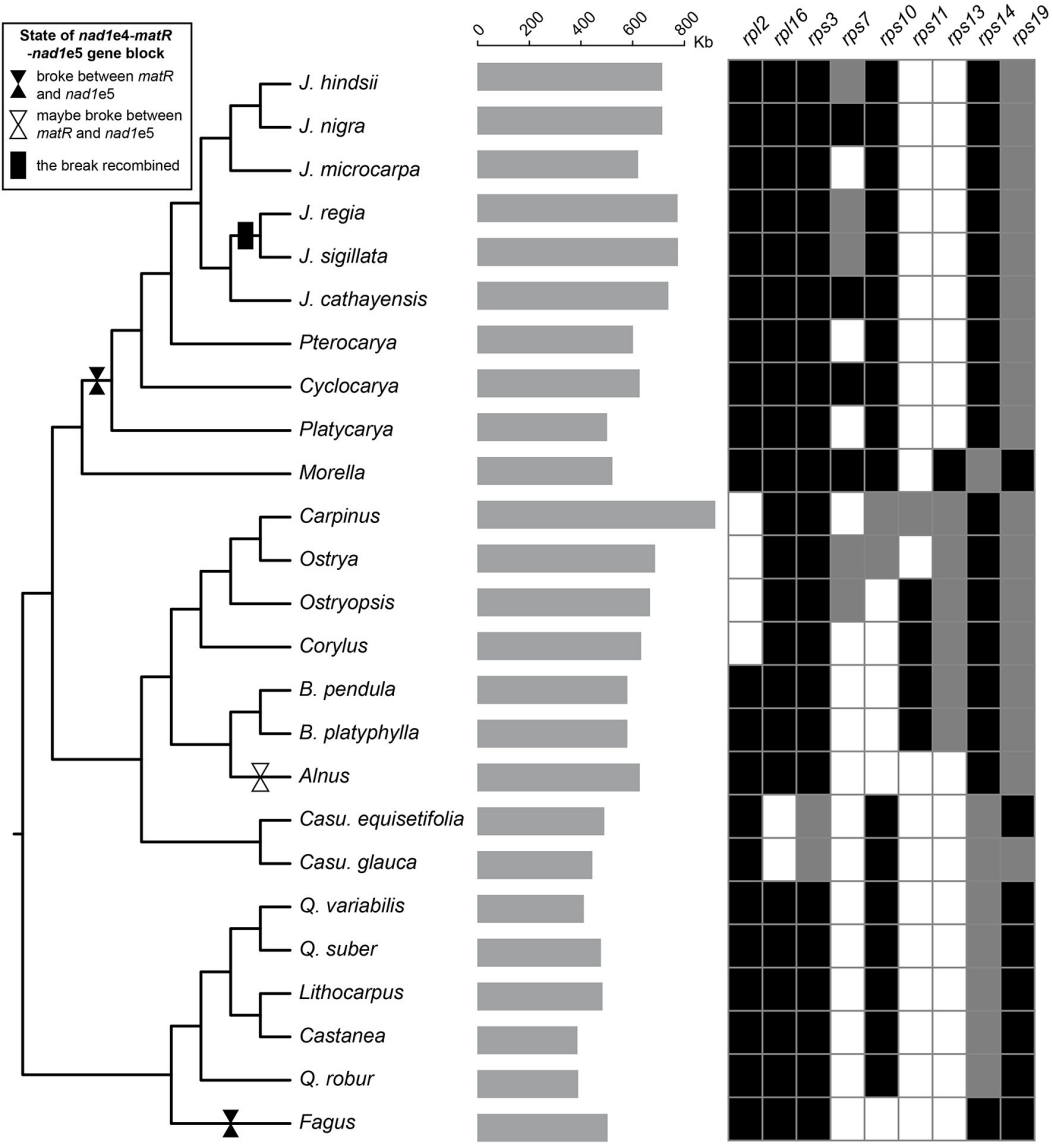
Sampling, mitogenome size, and gene variations. The gray bars in the middle showed the size of the mitogenomes. The grids on the right exhibit gene variations with black, gray, and blank indicating the gene intact, pseudo, and missing, respectively. The plastid tree was used to exhibit the species relationship. The breaks and reunion of the *nad1*e4-*matR*-*nad1*e5 block are marked on the branches.

**Figure 2:**
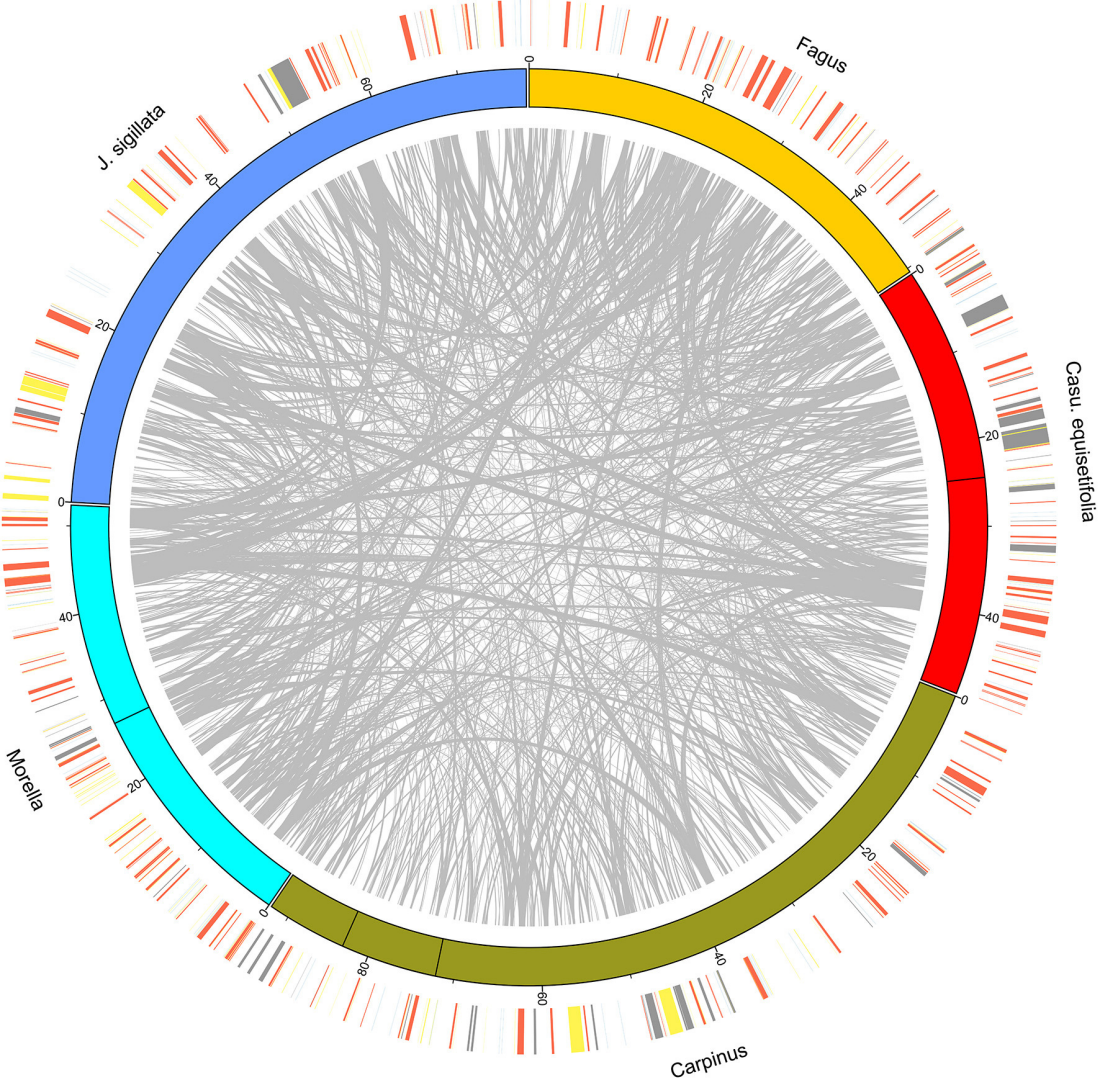
CIRCOS plot of species from five families. The longest mitogenomes of each family were used. The outer ring shows the position of protein-coding genes and rRNA (red), tRNA (blue), repeat (yellow), and MTPT (gray) sequences.

Characteristics of the mitogenome assemblies produced in this study, as well as previously published *B. pendula* and *Q. variabilis* assemblies, are provided in Table 1 and Figure 1. Mitogenome sizes in Casuarinaceae, Fagaceae, and Myricaceae resembled those of distant relatives from Rosales or Fabales (400 Kb and 480 Kb on average, respectively, NCBI data). By contrast, mitogenome sizes were substantially expanded in Betulaceae and Juglandaceae. The largest mitogenome was found in *Carpinus cordata* (922 Kb; Betulaceae) and was much larger than those of confamiliar species. Mitogenome sequences were less similar, and structures were highly rearranged, and many sequences have no homologs in other species, no matter between or within families (Figure 2 and Supplementary Figure 2).

The proportion of repeats in Fagales mitogenomes was small, typically <3% and no more than 6.2% of the total mitogenome length (Table 1). In Betulaceae, short repeats of <200 bp were more apparent, especially in *Alnus* (Supplementary Table 2). MTPT percentages were also low, with only two species having more than 6% (*Casu. equisetifolia*, 13.5%; and *Corylus*, ~9.5%).

The gene content of Fagales resembles other angiosperms. The 24 “core” protein-coding genes (*atp1, 4, 6, 8* and *9, ccmB, C, Fc* and *Fn, cob, cox1*-*3, nad1*-7, *9* and *4L, matR*, and *mttB*), three ribosomal RNA genes (*rrn5, rrnS*, and *rrnL*), and two succinate dehydrogenase subunit genes (*sdh3* and *sdh4*) are well preserved. As in many plants, the conservation of ribosomal protein genes is poor (Figure 1). Only 5 of them, *rpl5, rpl10, rps1, rps4*, and *rps12*, exist in all. Five of the seven Betulaceae species had *rps11* sequences with approximate identities of 100%. Comparison of Betulaceae *rps11* sequences with those in the NCBI *nt* database indicated similarities with *rps11* in monocots or basal core angiosperms such as *Triantha glutinosa* (KX808303, Alismatales) and *Liriodendron tulipifera* (NC_021152, Magnoliales), consistent with previous research (Bergthorsson et al., 2003). These similarities suggested that HGT of *rps11* may have occurred in a common Betulaceae ancestor, followed by differential losses in some species. Exon 4 of *nad1* (*nad1*e4), *matR*, and *nad1*e5 forms a colinear block in many angiosperms. This block was disrupted between *matR* and *nad1*e5 at least twice in Fagales species but, surprisingly, was recovered in *J. sigillata* and *J. regia* (Figure 1).

### Phylogenetic Relationship

**Figure 3:**
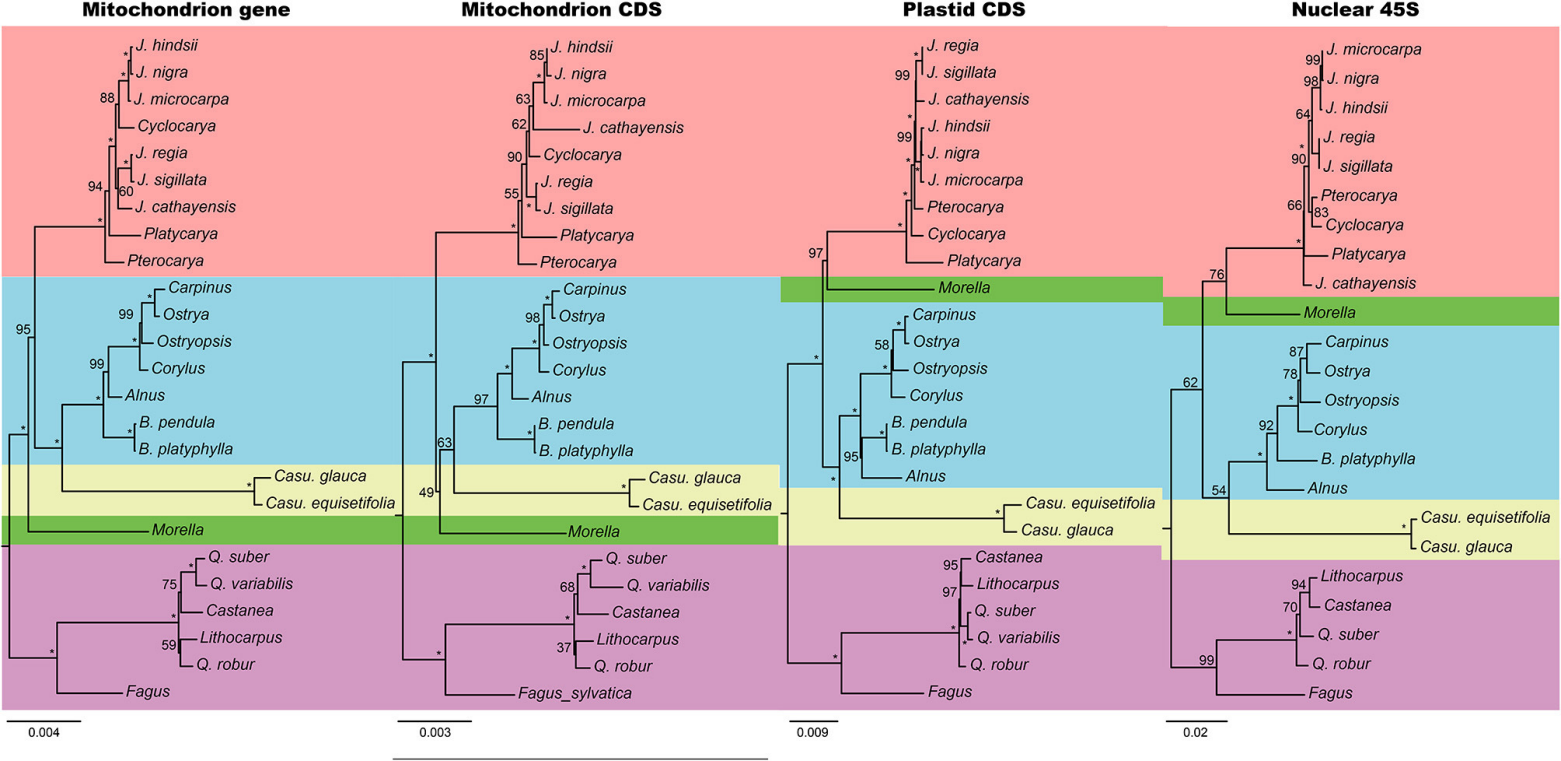
Phylogenetic trees reconstructed by mitochondrial genes, mitochondrial CDSs, plastid CDSs, and nuclear 45S. Numbers at nodes indicate the bootstrap support, and the full supports (100) were marked by asterisks. Each family used a different color as the background.

Phylogeny was reconstructed using four matrices from all the three genomes, namely, mitochondrial genes with introns (68,743 bp in length and 2,126 parsimony informative sites, PIS), mitochondrial CDSs without RNA-edited sites (31,551 bp and 750 PIS), plastid CDSs (69,243 bp and 6,495 PIS), and nuclear 45S (6,019 bp and 444 PIS). The trees of mitochondrial gene and plastid were robust, while those of mitochondrial CDS and nuclear were poorly supported (Figure 3). The nuclear tree was mostly congruent with the plastid, despite some nodes in Juglandaceae and Fagaceae. The most incongruence of the two trees was the position of Myricaceae, which was placed as the sister group of either “Betulaceae + Casuarinaceae” or “Betulaceae + Casuarinaceae + Juglandaceae”.

### Genus-Specific Sequences and Mosaic Origins

Repeat and MTPT sequences were not solely sufficient to explain the substantial size variation observed among mitogenomes from different species (Table 1). Furthermore, GSSs were identified and used to explore the causes of mitogenome size divergence. *Quercus* species were found to have nonmonophyletic relationships (Figure 3), and *Q. robur* was not included with other *Quercus* species when identifying *Quercus*-specific sequences. The GSSs and the total length of each species were given in Supplementary Tables 4, 6, respectively. As expected, *Juglans* yielded a large number of GSSs (105 Kb) since it has six species analyzed and their mitogenome size is generally bigger than those of relatives in the family. However, GSSs and mitogenome size showed a poor correlation in many other genera. *Casuarina*, which had relatively small mitogenomes and had the most GSSs (166 Kb). A similar situation was also observed in *Fagus* (105 Kb) and *Morella* (98 Kb). By contrast, *Carpinus*, which is the largest mitogenome and much longer than close relatives, did not contain correspondingly long GSSs (32 Kb).

**Figure 4:**
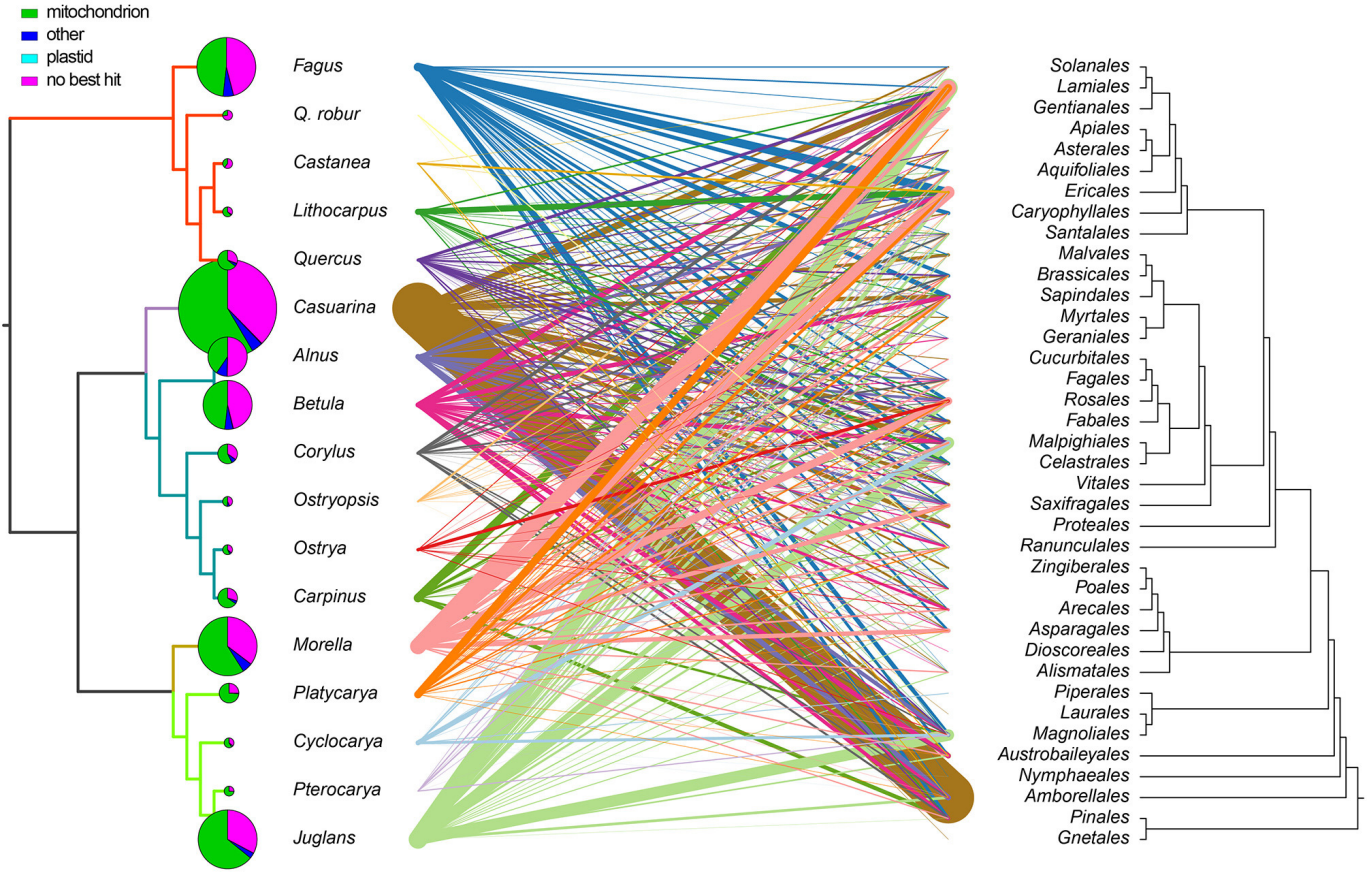
Analysis of mitogenomic GSSs. Best hits of GSSs between Fagales species and their hit taxa (combined into orders) are connected by lines. Each species is represented with a single color, and line thickness indicates the total sequence hit a length. Pie charts indicate the proportions of mitochondrial, plastid, and other hits, and the pie size represents the total GSS length. Details are in Supplementary Tables 4, 5.

Moreover, we searched these GSSs against NCBI *nt* database to detect the potential origins. Best matches of each region were retrieved and then grouped by compartment and order (Figure 4; Supplementary Tables 5, 6). Overall, the GSSs were related to a range of seed plant lineages and were mainly of mitogenomic origin (Figure 4). Some genera contained more best matches from certain orders, such as *Casuarina* from Amborellales; *Morella* from Lamiales and Ericales; *Juglans* from Lamiales, Malpighiales, and Magnoliales. Most of the GSSs and best matches were short, while some were quite long (Supplementary Figure 3).

### Identification of Other DNA

Mitochondrial plasmids are small autonomous circular or linear extrachromosomal DNA molecules in mitochondria, and these plasmids have been found in several species, including maize, rice, and carrot (McDermott et al., 2008). The origins and functions of mitochondrial plasmids remain unclear. In *Carpinus*, one 2,888-bp circular plasmid-like sequence was identified from the contigs. Its sequencing coverage resembled that of the mitogenome. Except for a small 240-bp plastid-like region, the circle had no sequence similarities with known angiosperm mitogenomes, including Fagales. It could be fully encompassed by *Carpinus avellana* or *Car. fangiana* nuclear sequences from different chromosomes. Its GC content was close to nuclear *Carpinus* genomes (*Car. fangiana*: 37.6%; Yang et al., 2020), but much lower than mtDNA (Table 1). Two open reading frames (ORFs), ORF244 (732 bp) and ORF162 (486 bp), could be predicted on the plasmid-like sequence. BLASTP comparison against the *nr* database identified homologs of ORF244 in several angiosperm species, including a nearly full-length match in *Arabidopsis thaliana* (AT1G74875, identical 34%). ORF244 homologs were annotated as putative F-box proteins, and homologs of ORF162 were annotated as DNA methylation four factors in several Rosids. It was unclear whether the two ORFs were expressed, but there was sufficient evidence to conclude that the sequence was of nuclear origin.

**Figure 5:**
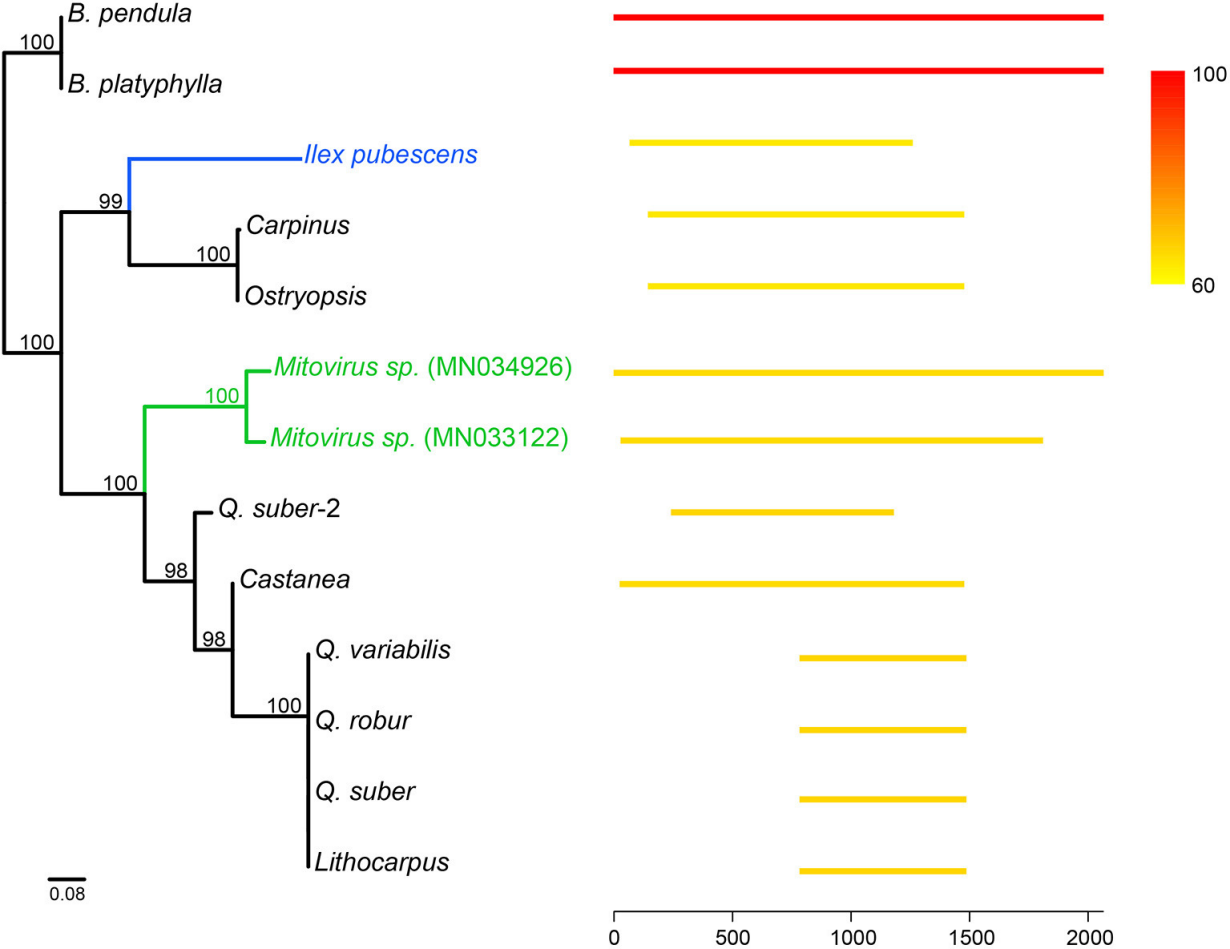
Mitovirus-like sequences in Fagales and other species. Long hits (>700 bp) were retrieved by searching the mitovirus-like sequence in *Betula* against NCBI *nt* database and other Fagales mitogenomes. At the right, lines show the position of the hits, and color indicates the similarity compared to the mitovirus-like sequence in *Betula* (red lines). The left tree was constructed by these hits using the ML method. The blue and green colors on the tree highlighted *Ilex* and mitoviruses, respectively.

Mitovirus-like sequences were also found in several Fagales. Mitoviruses, which belong to the Narnaviridae family, are positive single-stranded RNA viruses that replicate in host mitochondria. Mitovirus genomes are small, approximately 2.1–4.4 Kb in length, and contain a single ORF encoding a viral RNA-dependent RNA polymerase (RdRP) required for replication (Nibert, 2017). In *Betula*, a *ca*. 2-Kb region best matched two mitoviruses (GenBank: MN034926 and MN033122) in NCBI *nt* database. Behind the mitoviruses, it has another long hit from the mitogenome of *Ilex pubescens* (Aquifoliaceae, Asterids). In contrast, other hits were much shorter. Searching against Fagales mitogenomes, this region could get hits from many species. We used hits longer than 700 bp from these two databases to build the phylogeny (Figure 5). The tree revealed that these sequences were likely introduced into Fagales *via* multiple events.

Fagales belong to the nitrogen-fixing lineage of angiosperms, and at least three genera in this study have nitrogen-fixing capacity: *Casuarina, Morella*, and *Alnus* (Yelenik and D'Antonio, 2013; Huisman and Geurts, 2020). However, there was no indication that these genera contained sequences similar to bacteria.

## Discussion

### Assembling Plant Mitogenomes With Short Reads

Short reads can be used to retrieve plant mitogenomes that have been verified in many species. Their shortcomings are also apparent. Short reads could not overcome repeats longer than the sequencing read or insert length, resulting in fragmental contigs and artificial connections. From our experience and a brief survey, the quality of some known mitogenomes is concerning, including unfeasibly long repeats and MTPTs, inappropriate circularization, or having missing sequences, such as the absence of ribosomal RNA genes *rrnS* and *rrnL*. Those are challenging to reuse these data to get stringent conclusions. Our assembly method used in this study can obtain complete mitogenomes efficiently. Visual processes in the powerful software GENEIOUS allowed full verification of every base. The method might be not new and have similar versions used in other laboratories. However, we still want to highlight it here for the potential improvement of the future assemblies.

On the other hand, the structure of mitogenome *in vivo* is remaining mysterious. Despite the complex mitogenome structures observed under microscopy (Backert and Börner, 2000; Manchekar et al., 2006; Cheng et al., 2017), most mitogenomes can still be assembled as circles *in silico*. The connection underneath is unclear. Plant mitogenomes experience frequent rearrangements through their long repeats (Kozik et al., 2019), and it is thus unclear whether these mitogenomes can be considered to have a standard structure. The mitogenomes of *Fagus sylvatica* in the two independent projects were almost identical (this study and Mader et al., 2020), indicating preservation of mitogenomes among individuals in at least some plant species.

### Mitochondrial DNA and Phylogeny

We used four datasets to reconstruct the trees. The plastid tree is congruent with the previous study (Yang et al., 2021). In the matrix of nuclear 45S, most of the PISs are located in the internal transcribed spacers (ITSs) and seldom in rRNA genes. However, ITSs evolve quickly and some regions were difficult to align between families. That could be the reason for the low support of the nuclear tree. Mitochondrion, like plastid, may also reflect the evolution of the cytoplasm. Nevertheless, the capacity of mtDNA in phylogeny is yet to decide. Mitochondrial genes contain hundreds of RNA-edited sites (Small et al., 2020). These RNA-edited sites may interfere the tree building (Bowe and dePamphilis, 1996). A good way is to remove these sites. The poor support of mitochondrial CDSs without edited sites is mainly because of the low substitution rate (Palmer and Herbon, 1988), and the PISs were not enough. Although the mitochondrial genes, which include RNA-edited sites and introns, got a more robust tree, the incongruence between our two mitochondrial datasets may also challenge the ability of mitochondrial genes in phylogenetic construction in Fagales. For the noncoding regions, mitogenomes often convert DNA lesions into DBSs followed by inaccurate nonhomologous repairs (Gualberto and Newton, 2017; Christensen, 2018), which may introduce random mutations that mislead the tree building.

### Mitogenome Size Variation in Fagales

**Figure 6:**
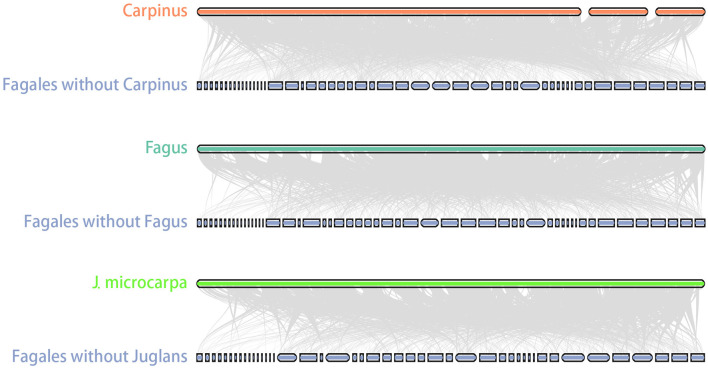
*Carpinus* mitogenome shared more homologous sequences with other Fagales. Homologous sequences between *Carpinus* and other Fagales mitogenomes were linked by gray lines. GSSs shared no homologous with others so that it would leave blank spaces that had no connections. *Carpinus* showed little blank spaces, and it proved that it has more homologous sequences with other Fagales. *Fagus* and *J. microcarpa* were used as comparisons.

Size variation between close species is a common feature of plant mitogenomes and has been observed in a range of taxa, such as *Viscum album* and *V. scurruloideum* (565 Kb *vs*. 66 Kb; Petersen et al., 2015; Skippington et al., 2015), *Silene conica* and *S. noctiflora* (11.1 Mb vs. 6.7 Mb; Wu et al., 2015; Wu and Sloan, 2018), and *Cucumis melo* and *C. sativus* (2.7 Mb vs. 1.7 Mb; Alverson et al., 2011; Rodríguez-Moreno et al., 2011). The reasons for this size variability may be complex. Duplications, intracellular transfer events, and introductions of foreign DNA all contribute to mitogenome size expansion (Alverson et al., 2011; Rice et al., 2013). In Fagales, the mitogenome of *Carpinus* is notably larger than those of close relatives. However, lengths of repeats, MTPTs, and GSSs were insufficient to explain the size divergence. Another possibility is that the *Carpinus* mitogenome has an unusually high number of homologs with other Fagales, which was confirmed by the homolog searches between *Carpinus* and other Fagales (Figure 6). Most interestingly, it raises the question of what was the ancestral mitogenome like in Fagales. One potential is that the ancestral mitogenome was similarly as large as that of *Carpinus*, and sequences were then lost independently in different lineages during evolution. This model was used to explain the mitogenome size variation in kiwifruits (Wang et al., 2019). However, it appears unlikely that all Fagales genera other than *Carpinus* experienced such large and variable sequence losses, suggesting that sequence transfer may be a more likely scenario for Fagales.

Intracellular DNA transfer between genome compartments is a common phenomenon. Interactions between nuclear and mitochondrial genomes may occur frequently and contribute significantly to mitogenome size (Alverson et al., 2011; Goremykin et al., 2012). Although we attempted to analyze the nuclear insertions in the mitogenomes, our efforts did not yield, because Fagales nuclear genomes seem all to contain mitochondrial contigs. In this case, it is challenging to get accurate results. Instead, the plasmid-like sequence of nuclear origin was found in *Carpinus*, which could reflect that its mitogenome has integrated nuclear insertions. The mitochondrial plasmid may be an intermediary stage prior to incorporation into the chromosomal mitogenome.

### Mosaic Evolution of Mitogenomes

Our GSS analysis showed that Fagales mitogenomes exhibited mosaic characteristics, which could be a general feature of all seed plant mitogenomes. Plant mitogenomes are prone to absorb foreign DNA, but this ability also has its limits. “Mitochondrial fusion occurs in a fundamentally similar manner” (Rice et al., 2013), so that plant mitogenomes are easier to get DNA from other plants. Our results complied with this rule as no sequences appeared to be derived from other cellular organisms out of seed plants, even though some species were symbionts with nitrogen-fixing bacteria. Some of these GSSs are likely horizontally transferred, especially those orders that are distantly related to Fagales but received large amounts of best hits, such as *Amborella*. A previous study has shown that *Amborella* contains HGTs from many species, including Fagales (Rice et al., 2013). We found that these HGT-like sequences were mainly shared with *Casuarina*. As we used GSSs in the analysis, the direction of them was undecided. The “wounding-HGT model” could explain massive HGTs between nonparasitic plants (Rice et al., 2013). In comparison, this model seems unconvincing if applied to the widespread mosaic sequences in land plants.

Hints may come from the mitovirus-like sequences in this study. Mitovirus sequences, particularly those corresponding to the RdRP region, are widespread in plant nuclear and mitochondrial genomes (Alverson et al., 2011; Bruenn et al., 2015; Nibert, 2017; Silva et al., 2017; Chu et al., 2018; Nibert et al., 2018; Charon et al., 2020). Plant mitovirus-like sequences were thought to be derived from plant pathogenic fungal interactions and HGT events (Bruenn et al., 2015). However, direct HGT from fungal to plant mitogenomes is unlikely, as incompatibility hampers fusion between mitochondria in fungi and plants (Rice et al., 2013). An alternative path is transferring from fungi to the plant nuclear genome, and then from the nucleus to the plant mitogenome. This idea was also excluded by searching the mitovirus-like sequence against *B. nana* and *B. pendula* nuclear genomes (Wang et al., 2013; Salojärvi et al., 2017). It is therefore possible that mitoviruses can infect plants directly and frequently (Figure 5; Vong et al., 2019). The mitovirus-like sequence found in *Ilex pubescens* could also be another independent infection instead of HGT from Fagales.

**Figure 7:**
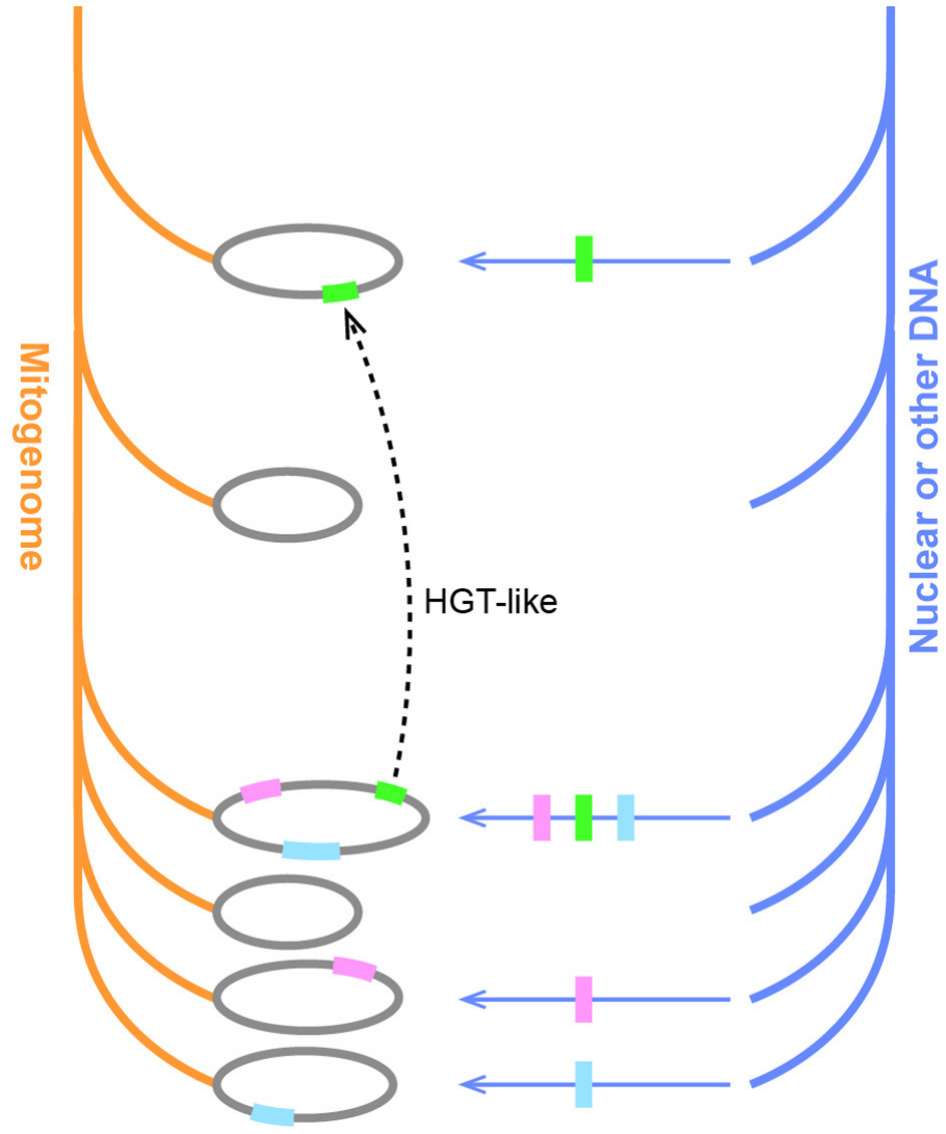
The hypothesis of the mosaic evolution in plant mitogenomes. Orange and blue branches represent the inheritance of mitogenome and other DNA, respectively. Horizontal arrows indicate DNA transfers, and colorful blocks indicate different sequences. Unequal transfers result in some species acquiring additional homologs. The dotted line indicates the creation of an HGT-like sequence upon the transfer of a single sequence on two independent occasions in distant lineages.

In conclusion, the “third-party” DNA, including mitovirus and nuclear insertions, may account partially for the mosaic composition of plant mitogenomes. The mosaic HGT-like sequences in angiosperm mitogenomes may be similar underneath to the expanded set of homologs observed in *Carpinus*. If two species get DNA from the same source, we sometimes can make an illusion that similar sequences are shared with far-away lineages; if different dosages were transferred in independent events, some species may share more homologs with others (Figure 7). Since the transfers between the third parties and mitogenomes could happen independently and were not limited to time, and mitogenomes themselves also encountered continuous rearrangements and deletion, from time to time it would finally create extremely mosaic mitogenomes.

## Data Availability Statement

The assembled sequences have been deposited to CNGB Sequence Archive of China National GeneBank DataBase (CNGBdb, https://db.cngb.org/) under Project CNP0001491 (mitogenomes: accessions N_000011064 - N_000011115; plastomes: accessions N_000011061 - N_000011063; *Carpinus* mitochondrial plasmid-like sequence: accession N_000011116).

## Code Availability

The used scripts can be found in Github (https://github.com/fengyanlei33/Fagales_mitogenome).

## Author Contributions

YF, ZF, and XJ designed the project. YF assembled and annotated the genomes. YF, XX, DA, RP, ZF, and XJ worked together to finish the analyses and the manuscript. All authors contributed to the article and approved the submitted version.

## Funding

This work was supported by the National Natural Science Foundation of China (Grant no. 32000158), the National Key R&D Program of China (Grant no. 2019YFA0906300), the Project of Sustainable Development Research Center of Resources and Environment of Western Sichuan, Sichuan Normal University (Grant no. 2020CXZX03), the Leading Innovative and Entrepreneur Team Introduction Program of Zhejiang (Grant no. 2019R01002), and Westlake Postdoc Project (Grant no. 101196582003).

## Conflict of Interest

The authors declare that the research was conducted in the absence of any commercial or financial relationships that could be construed as a potential conflict of interest.

## Publisher's Note

All claims expressed in this article are solely those of the authors and do not necessarily represent those of their affiliated organizations, or those of the publisher, the editors and the reviewers. Any product that may be evaluated in this article, or claim that may be made by its manufacturer, is not guaranteed or endorsed by the publisher.

## References

[B1] Alverson A. J. Rice D. W. Dickinson S. Barry K. Palmer J. D. (2011). Origins and recombination of the bacterial-sized multichromosomal mitochondrial genome of cucumber. Plant Cell. 23, 2499–2513. 10.1105/tpc.111.087189 21742987 PMC3226218

[B2] Backert S. Börner T. (2000). Phage T4-like intermediates of DNA replication and recombination in the mitochondria of the higher plant Chenopodium album (L.). Curr. Genet. 37, 304–314. 10.1007/s002940050532 10853767

[B3] Bankevich A. Nurk S. Antipov D. Gurevich A. A. Dvorkin M. Kulikov A. S.. (2012). SPAdes: a new genome assembly algorithm and its applications to single-cell sequencing. J. Comput. Biol. 19, 455–477. 10.1089/cmb.2012.0021 22506599 PMC3342519

[B4] Bergthorsson U. Adams K. L. Thomason B. Palmer J. D. (2003). Widespread horizontal transfer of mitochondrial genes in flowering plants. Nature. 424, 197. 10.1038/nature01743 12853958

[B5] Bi Q. Li D. Zhao Y. Wang M. Li Y. Liu X.. (2019). Complete mitochondrial genome of Quercus variabilis (Fagales, Fagaceae). Mitochondrial DNA Part B. 4, 3927–3928. 10.1080/23802359.2019.1687027 33366255 PMC7707397

[B6] Bolger A. M. Lohse M. Usadel B. (2014). Trimmomatic: a flexible trimmer for Illumina sequence data. Bioinformatics. 30, 2114–2120. 10.1093/bioinformatics/btu170 24695404 PMC4103590

[B7] Bowe L. M. dePamphilis C. W. (1996). Effects of RNA editing and gene processing on phylogenetic reconstruction. Mol. Biol. Evol. 13, 1159–1166. 10.1093/oxfordjournals.molbev.a025680 8896368

[B8] Bruenn J. A. Warner B. E. Yerramsetty P. (2015). Widespread mitovirus sequences in plant genomes. PeerJ. 3, e876. 10.7717/peerj.876 25870770 PMC4393810

[B9] Camacho C. Coulouris G. Avagyan V. Ma N. Papadopoulos J. Bealer K.. (2009). BLAST plus: architecture and applications. BMC Bioinformatics. 10. 10.1186/1471-2105-10-421 20003500 PMC2803857

[B10] Chan P. P. Lowe T. M. (2019). tRNAscan-SE: Searching for tRNA Genes in Genomic Sequences. Methods. Mol. Biol. 1962, 1–14. 10.1007/978-1-4939-9173-0_1 31020551 PMC6768409

[B11] Charon J. Marcelino V. R. Wetherbee R. Verbruggen H. Holmes E. C. (2020). Meta-transcriptomic detection 1 of diverse and divergent RNA viruses in green and chlorarachniophyte algae. bioRxiv [Preprint]. 10.1101/2020.06.08.141184 PMC7594059 33086653

[B12] Cheng N. Lo Y. S. Ansari M. I. Ho K. C. Jeng S. T. Lin N. S.. (2017). Correlation between mtDNA complexity and mtDNA replication mode in developing cotyledon mitochondria during mung bean seed germination. New Phytol. 213, 751–763. 10.1111/nph.14158 27611966

[B13] Christensen A. C. (2018). Mitochondrial DNA Repair and Genome *Evolution*. 11–32. 10.1002/9781119312994.apr054423645599

[B14] Chu H. Jo Y. Choi H. Lee B. C. Cho W. K. (2018). Identification of viral domains integrated into Arabidopsis proteome. Mol. Phylogenet. Evol. 128, 246–257. 10.1016/j.ympev.2018.08.009 30125655

[B15] Goremykin V. V. Lockhart P. J. Viola R. Velasco R. (2012). The mitochondrial genome of Malus domestica and the import-driven hypothesis of mitochondrial genome expansion in seed plants. Plant J. 71, 615–626. 10.1111/j.1365-313X.2012.05014.x 22469001

[B16] Griesmann M. Chang Y. Liu X. Song Y. Haberer G. Crook M. B.. (2018). Phylogenomics reveals multiple losses of nitrogen-fixing root nodule symbiosis. Science. 361, eaat1743. 10.1126/science.aat1743 29794220

[B17] Gualberto J. M. Newton K. J. (2017). Plant Mitochondrial Genomes: Dynamics and Mechanisms of Mutation. Annu. Rev. Plant. Biol. 68, 225–252. 10.1146/annurev-arplant-043015-112232 28226235

[B18] Huisman R. Geurts R. (2020). A Roadmap toward Engineered Nitrogen-Fixing Nodule Symbiosis. Plant Commun. 1, 100019. 10.1016/j.xplc.2019.100019 33404552 PMC7748023

[B19] Jia H. M. Jia H. J. Cai Q. L. Wang Y. Zhao H. B. Yang W. F.. (2019). The red bayberry genome and genetic basis of sex determination. Plant Biotechnol J. 17, 397–409. 10.1111/pbi.12985 29992702 PMC6335074

[B20] Katoh K. Standley D. M. (2013). MAFFT Multiple Sequence Alignment Software Version 7: Improvements in Performance and Usability. Mol Biol. Evol. 30, 772–780. 10.1093/molbev/mst010 23329690 PMC3603318

[B21] Kozik A. Rowan B. A. Lavelle D. Berke L. Schranz M. E. Michelmore R. W.. (2019). The alternative reality of plant mitochondrial DNA: One ring does not rule them all. PLoS Genet. 15, e1008373. 10.1371/journal.pgen.1008373 31469821 PMC6742443

[B22] Krzywinski M. Schein J. Birol I. Connors J. Gascoyne R. Horsman D.. (2009). Circos: an information aesthetic for comparative genomics. Genome Res. 19, 1639–1645. 10.1101/gr.092759.109 19541911 PMC2752132

[B23] Lovell J. T. Bentley N. B. Bhattarai G. Jenkins J. W. Sreedasyam A. Alarcon Y.. (2021). Four chromosome scale genomes and a pan-genome annotation to accelerate pecan tree breeding. Nat Commun. 12, 4125. 10.1038/s41467-021-24328-w 34226565 PMC8257795

[B24] Lucas S. J. Kahraman K. Avsar B. Buggs R. J. A. Bilge I. (2021). A chromosome-scale genome assembly of European hazel (Corylus avellana L.) reveals targets for crop improvement. Plant J. 105, 1413–1430. 10.1111/tpj.15099 33249676

[B25] Mader M. Schroeder H. Schott T. Schoning-Stierand K. Leite Montalvao A. P. Liesebach H.. (2020). Mitochondrial genome of Fagus sylvatica L. as a source for taxonomic marker development in the fagales. Plants (Basel). 9, 1274. 10.3390/plants9101274 32992588 PMC7650814

[B26] Manchekar M. Scissum-Gunn K. Song D. Khazi F. McLean S. L. Nielsen B. L. (2006). DNA recombination activity in soybean mitochondria. J. Mol. Biol. 356, 288–299. 10.1016/j.jmb.2005.11.070 16376379

[B27] McDermott P. Connolly V. Kavanagh T. A. (2008). The mitochondrial genome of a cytoplasmic male sterile line of perennial ryegrass (Lolium perenne L.) contains an integrated linear plasmid-like element. Theor. Appl. Genet. 117, 459–470. 10.1007/s00122-008-0790-7 18504541

[B28] Mower J. P. (2009). The PREP suite: predictive RNA editors for plant mitochondrial genes, chloroplast genes and user-defined alignments. Nucleic. Acids. Res. 37, W253–259. 10.1093/nar/gkp337 19433507 PMC2703948

[B29] Mower J. P. Sloan D. B. Alverson A. J. (2012). Plant Mitochondrial Genome Diversity: The Genomics *Revolution*. 123–144. 10.1007/978-3-7091-1130-7_9

[B30] Nguyen L. T. Schmidt H. A. von Haeseler A. Minh B. Q. (2015). IQ-TREE: a fast and effective stochastic algorithm for estimating maximum-likelihood phylogenies. Mol. Biol. Evol. 32, 268–274. 10.1093/molbev/msu300 25371430 PMC4271533

[B31] Nibert M. L. (2017). Mitovirus UGA(Trp) codon usage parallels that of host mitochondria. Virology. 507, 96–100. 10.1016/j.virol.2017.04.010 28431284 PMC5517309

[B32] Nibert M. L. Vong M. Fugate K. K. Debat H. J. (2018). Evidence for contemporary plant mitoviruses. Virology. 518, 14–24. 10.1016/j.virol.2018.02.005 29438872 PMC6668999

[B33] Palmer J. D. Herbon L. A. (1988). Plant mitochondrial DNA evolves rapidly in structure, but slowly in sequence. J. Mol. Evol. 28, 87–97. 3148746 10.1007/BF02143500

[B34] Paradis E. Claude J. Strimmer K. (2004). APE: Analyses of Phylogenetics and Evolution in R language. Bioinformatics. 20, 289–290. 10.1093/bioinformatics/btg412 14734327

[B35] Petersen G. Cuenca A. Moller I. M. Seberg O. (2015). Massive gene loss in mistletoe (Viscum, Viscaceae) mitochondria. Sci. Rep. 5, 17588. 10.1038/srep17588 26625950 PMC4667250

[B36] Rice D. W. Alverson A. J. Richardson A. O. Young G. J. Sanchez-Puerta M. V. Munzinger J.. (2013). Horizontal transfer of entire genomes via mitochondrial fusion in the angiosperm amborella. Science. 342, 1468–73. 10.1126/science.1246275 24357311

[B37] Rodríguez-Moreno L. González V. M. Benjak A. Mart,í M. C. Puigdomènech P. Aranda M. A.. (2011). Determination of the melon chloroplast and mitochondrial genome sequences reveals that the largest reported mitochondrial genome in plants contains a significant amount of DNA having a nuclear origin. BMC Genomics. 12, 424–424. 10.1186/1471-2164-12-424 21854637 PMC3175227

[B38] Salojärvi J. Smolander O.-P. Nieminen K. Rajaraman S. Safronov O. Safdari P.. (2017). Genome sequencing and population genomic analyses provide insights into the adaptive landscape of silver birch. Nat. Genet. 49, 904–912. 10.1038/ng.3862 28481341

[B39] Sennikov A. N. Soltis D. E. Mabberley D. J. Byng J. W. Fay M. F. Christenhusz M. J. M.. (2016). An update of the Angiosperm Phylogeny Group classification for the orders and families of flowering plants: APG IV. Bot J Linn Soc. 181, 1–20. 10.1111/boj.12385

[B40] Silva S. R. Alvarenga D. O. Aranguren Y. Penha H. A. Fernandes C. C. Pinheiro D. G.. (2017). The mitochondrial genome of the terrestrial carnivorous plant Utricularia reniformis (Lentibulariaceae): Structure, comparative analysis and evolutionary landmarks. Plos ONE. 12, e0180484. 10.1371/journal.pone.0180484 28723946 PMC5516982

[B41] Skippington E. Barkman T. J. Rice D. W. Palmer J. D. (2015). Miniaturized mitogenome of the parasitic plant Viscum scurruloideum is extremely divergent and dynamic and has lost all nad genes. P. Natl. Acad. Sci. USA. 112, E3515–E3524. 10.1073/pnas.1504491112 26100885 PMC4500244

[B42] Sloan D. B. Alverson A. J. Chuckalovcak J. P. Wu M. McCauley D. E. Palmer J. D.. (2012). Rapid evolution of enormous, multichromosomal genomes in flowering plant mitochondria with exceptionally high mutation rates. PLoS Biol. 10, e1001241. 10.1371/journal.pbio.1001241 22272183 PMC3260318

[B43] Small I. D. Schallenberg-Rudinger M. Takenaka M. Mireau H. Ostersetzer-Biran O. (2020). Plant organellar RNA editing: what 30 years of research has revealed. Plant J. 101, 1040–1056. 10.1111/tpj.14578 31630458

[B45] Tang H. Bowers J. E. Wang X. Ming R. Alam M. Paterson A. H. (2008). Synteny and collinearity in plant genomes. Science. 320, 486–488. 10.1126/science.1153917 18436778

[B46] Vong M. Manny A. R. Smith K. L. Gao W. Nibert M. L. (2019). Beta vulgaris mitovirus 1 in diverse cultivars of beet and chard. Virus Res. 265, 80–87. 10.1016/j.virusres.2019.02.008 30853586 PMC6668331

[B47] Wang N. Thomson M. Bodles W. J. Crawford R. M. Hunt H. V. Featherstone A. W.. (2013). Genome sequence of dwarf birch (Betula nana) and cross-species RAD markers. Mol. Ecol. 22, 3098–3111. 10.1111/mec.12131 23167599

[B48] Wang S. Li D. Yao X. Song Q. Wang Z. Zhang Q.. (2019). Evolution and Diversification of Kiwifruit Mitogenomes through Extensive Whole-Genome Rearrangement and Mosaic Loss of Intergenic Sequences in a Highly Variable Region. Genome. Biol. Evol. 11, 1192–1206. 10.1093/gbe/evz063 30895302 PMC6482417

[B49] Wolfe K. H. Li W. H. Sharp P. M. (1987). Rates of nucleotide substitution vary greatly among plant mitochondrial, chloroplast, and nuclear DNAs. Proc. Natl. Acad. Sci. U S A. 84, 9054–9058. 10.1073/pnas.84.24.9054 3480529 PMC299690

[B50] Wu Z. Cuthbert J. M. Taylor D. R. Sloan D. B. (2015). The massive mitochondrial genome of the angiosperm Silene noctiflora is evolving by gain or loss of entire chromosomes. Proc. Natl. Acad. Sci. U S A. 112, 10185–10191. 10.1073/pnas.1421397112 25944937 PMC4547255

[B51] Wu Z. Sloan D. B. (2018). Recombination and intraspecific polymorphism for the presence and absence of entire chromosomes in mitochondrial genomes. Heredity (Edinb). 122, 647–659. 10.1038/s41437-018-0153-3 30356223 PMC6461862

[B52] Wynn E. L. Christensen A. C. (2018). Repeats of unusual size in plant mitochondrial genomes: identification, incidence and evolution. G3 (Bethesda). 9, 549–59. 10.1534/g3.118.200948 30563833 PMC6385970

[B53] Xiang X.-G. Wang W. Li R.-Q. Lin L. Liu Y. Zhou Z.-K.. (2014). Large-scale phylogenetic analyses reveal fagalean diversification promoted by the interplay of diaspores and environments in the Paleogene. Perspect. Plant. Ecol. Evol. Syst. 16, 101–110. 10.1016/j.ppees.2014.03.001

[B54] Yang X. Wang Z. Zhang L. Hao G. Liu J. Yang Y. (2020). A chromosome-level reference genome of the hornbeam, Carpinus fangiana. Sci. Data. 7, 24. 10.1038/s41597-020-0370-5 31964866 PMC6972722

[B55] Yang Y. Y. Qu X. J. Zhang R. Stull G. W. Yi T. S. (2021). Plastid phylogenomic analyses of Fagales reveal signatures of conflict and ancient chloroplast capture. Mol. Phylogenet. Evol. 163, 107232. 10.1016/j.ympev.2021.107232 34129935

[B56] Yelenik S. G. D'Antonio C. M. (2013). Self-reinforcing impacts of plant invasions change over time. Nature. 503, 517–520. 10.1038/nature12798 24256723

