# Supplementary figures and images for: Mitochondrial Phylogenomics of Fagales Provides Insights Into Plant Mitogenome Mosaic Evolution
Source: Front Plant Sci. 2021 Oct 18;12:762195. doi: 10.3389/fpls.2021.762195 (PMC8558628; doi:10.3389/fpls.2021.762195)

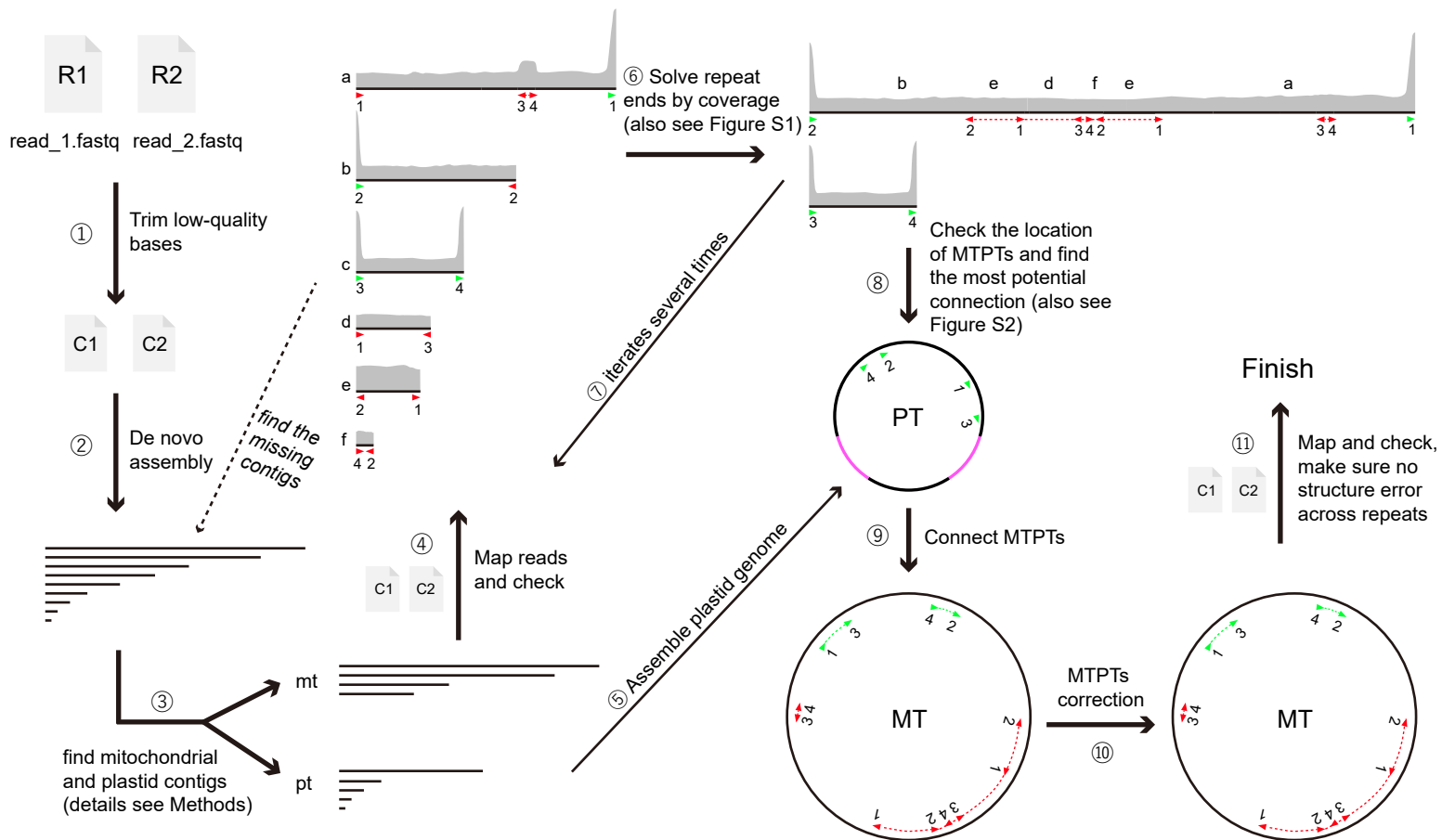

Supplement: Supplementary file 1 [file Data_Sheet_1.PDF]

## Betulaceae

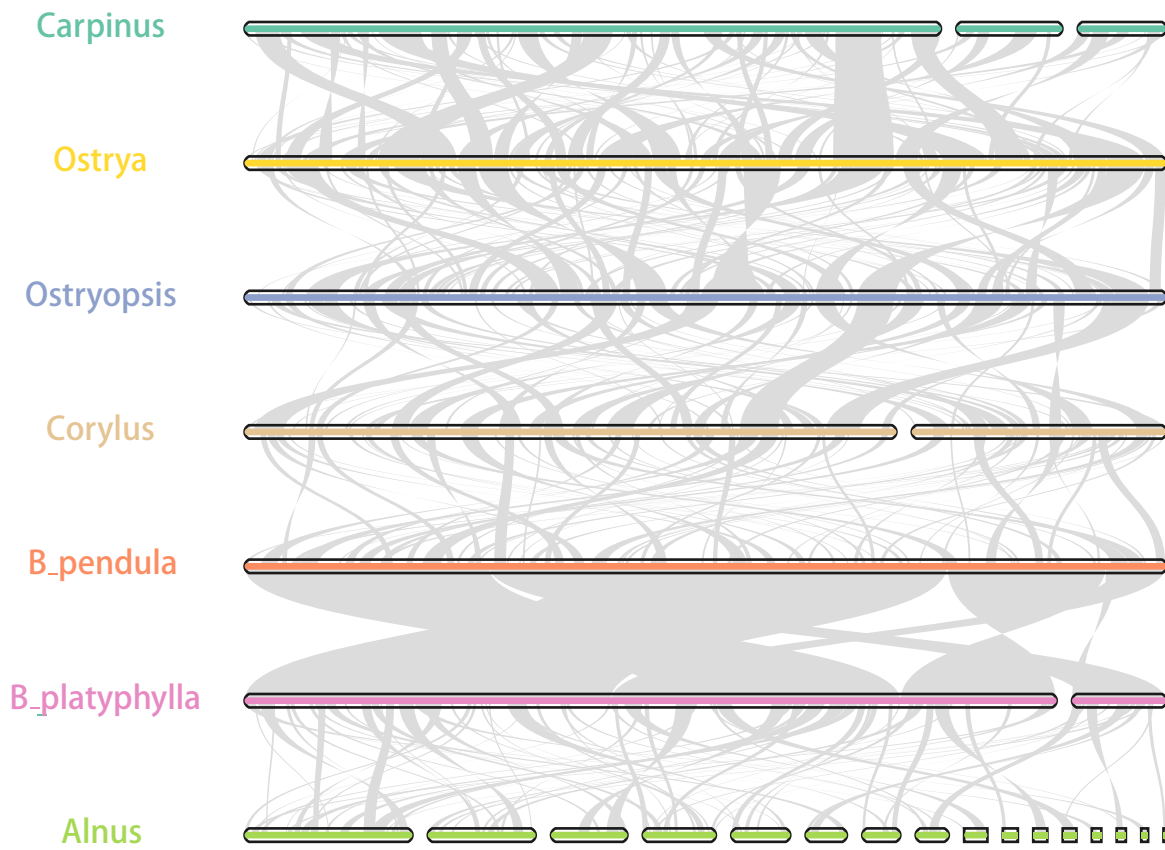

## Casuarinaceae

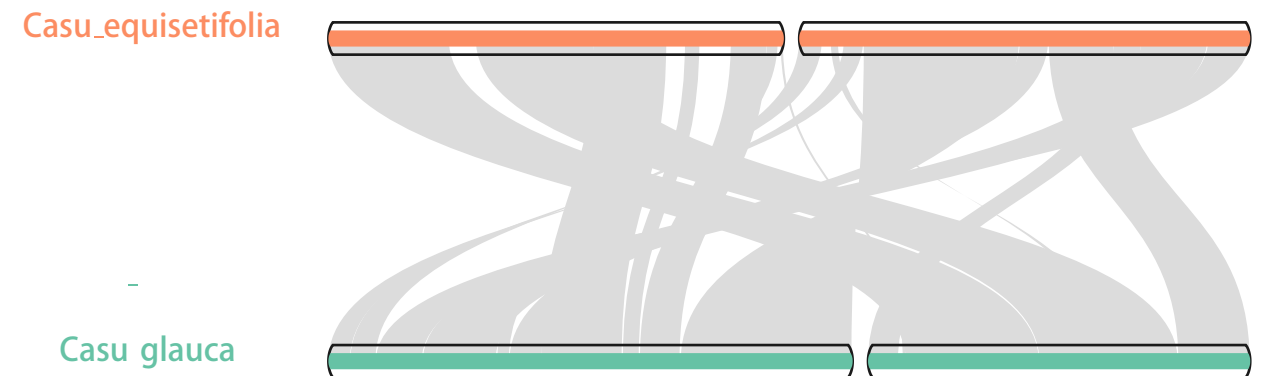

## Juglandaceae

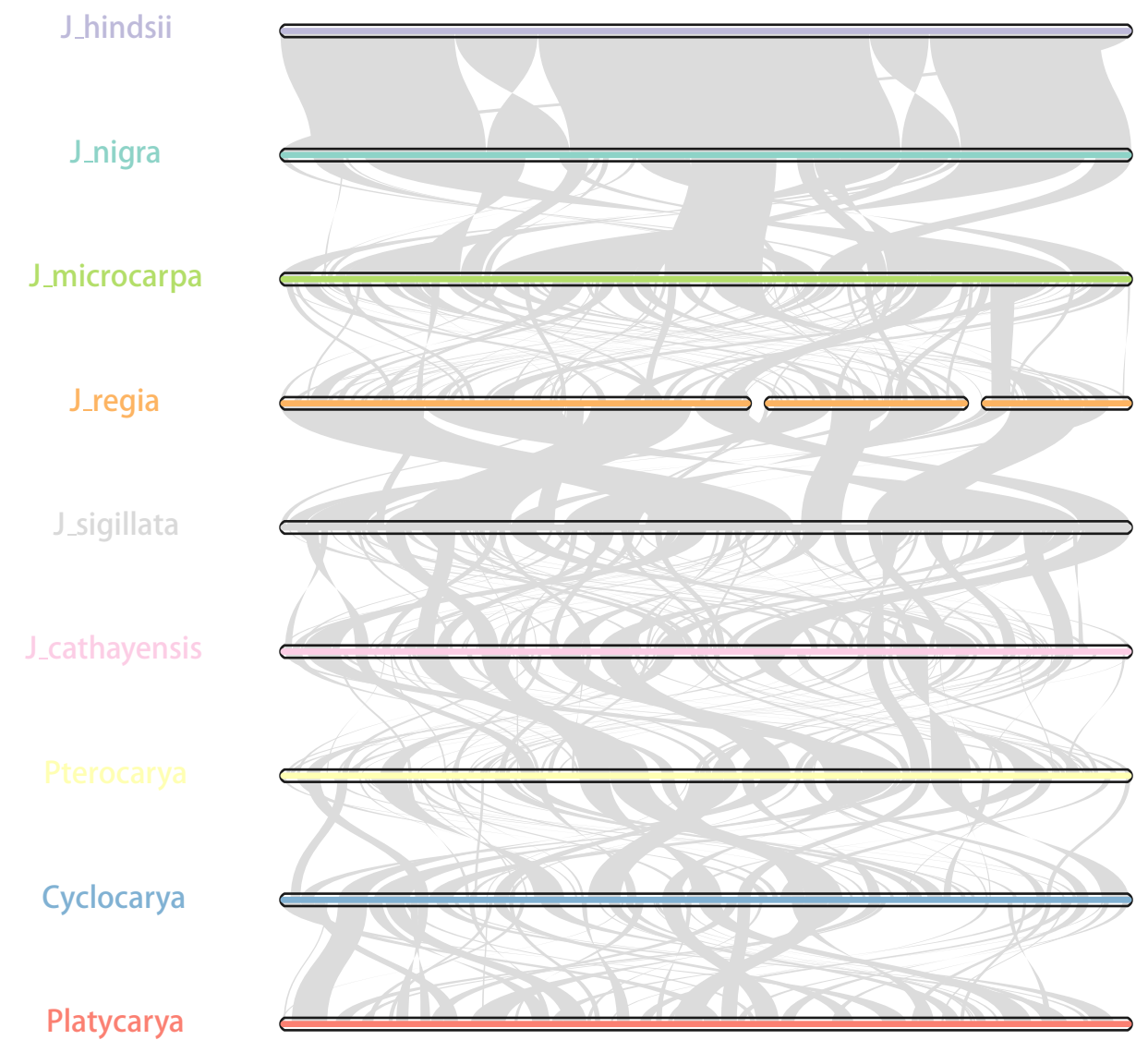

## Fagaceae

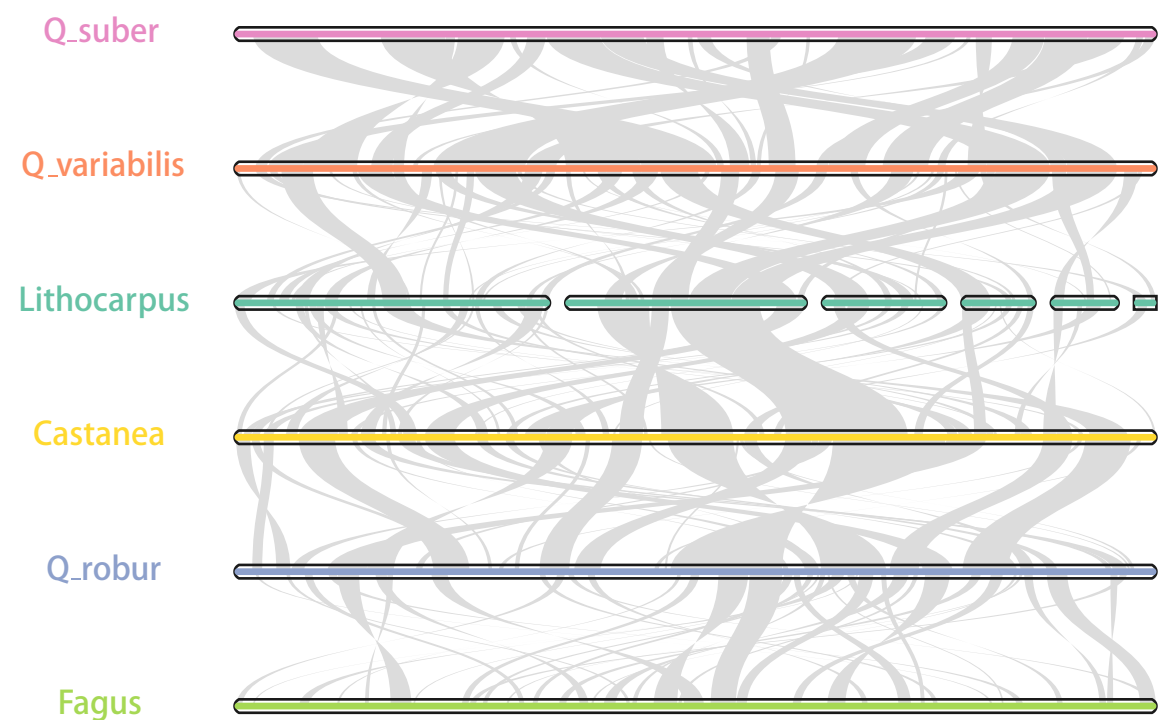

Supplement: Supplementary file 2 [file Data_Sheet_2.PDF]

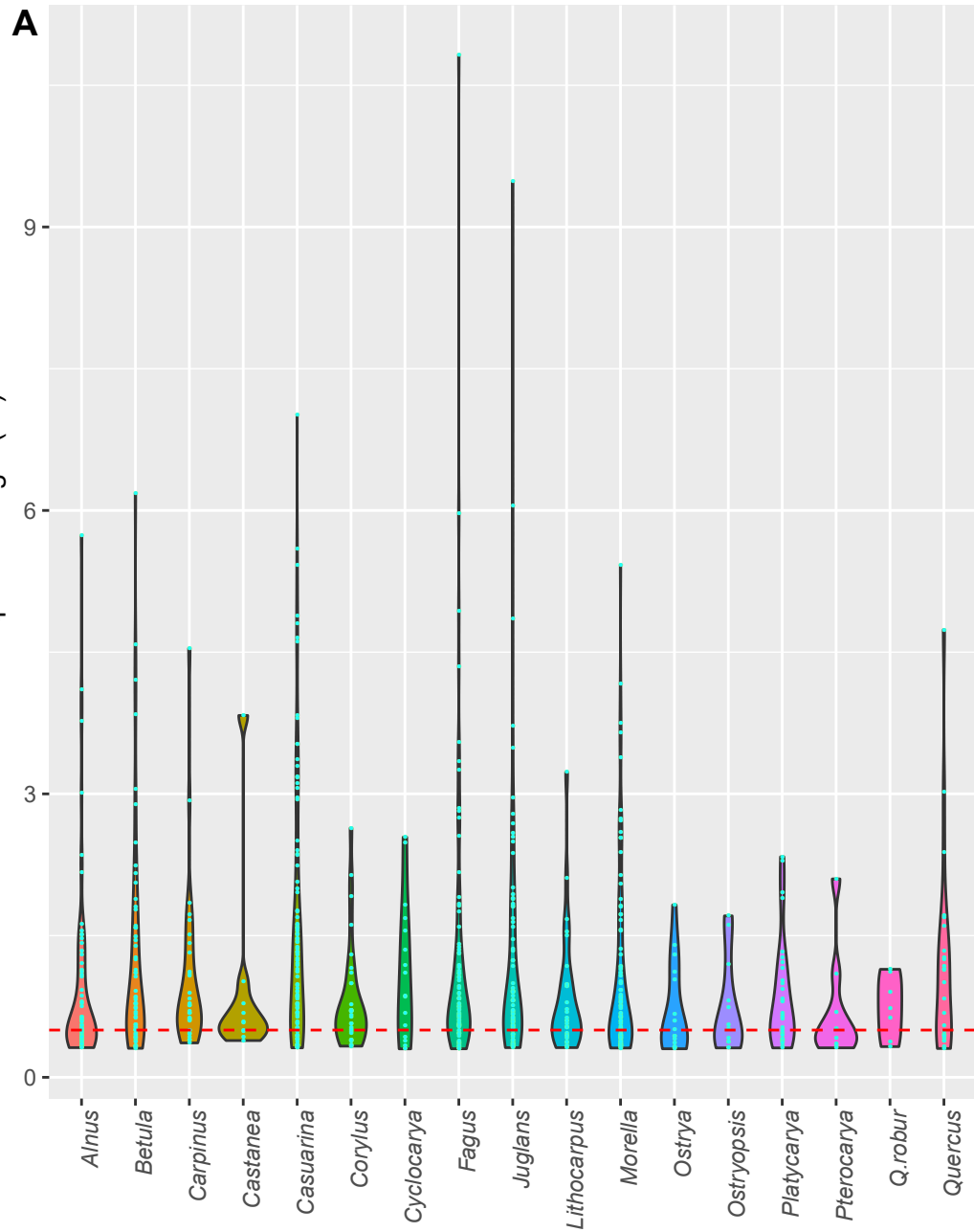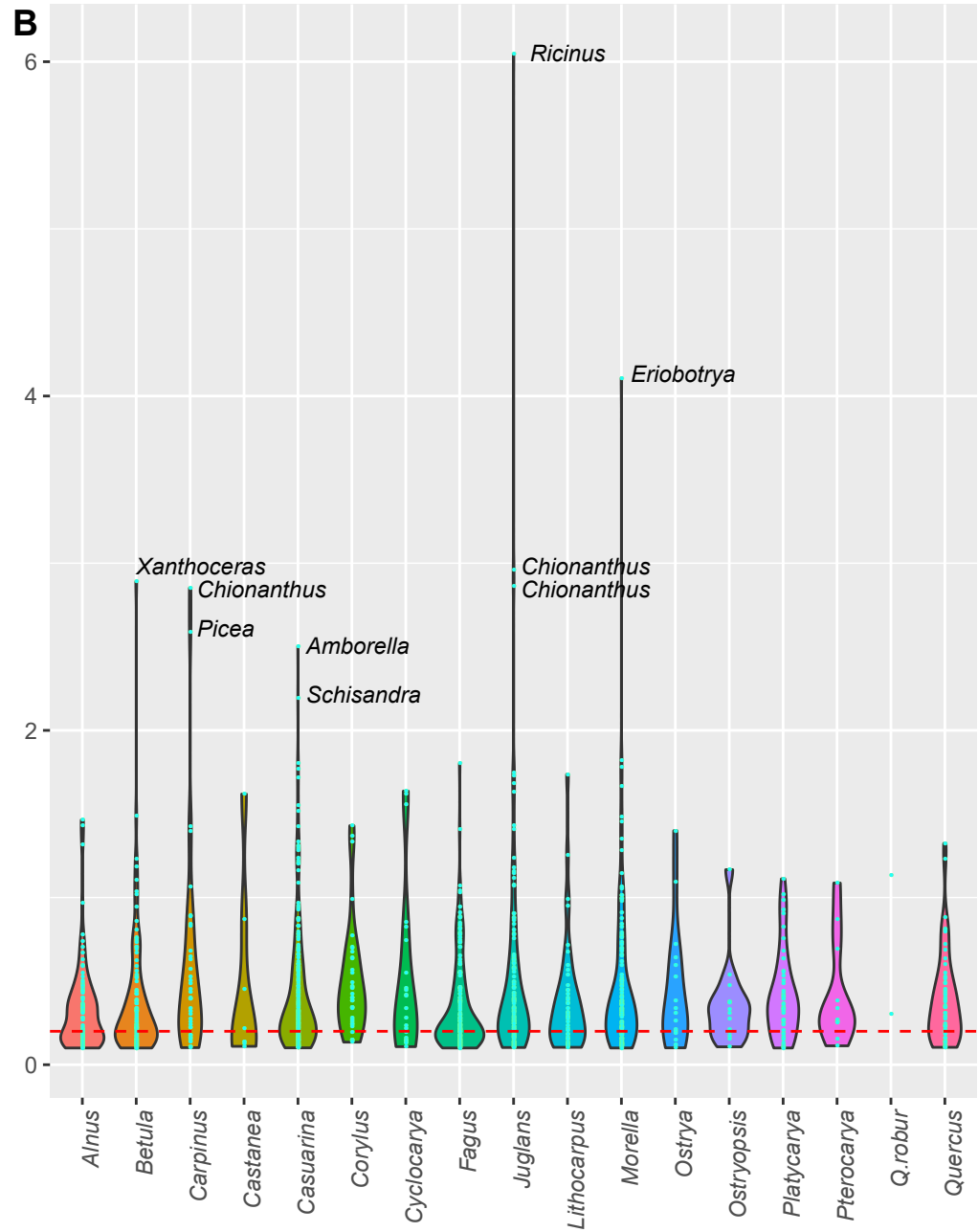

Supplement: Supplementary file 3 [file Data_Sheet_3.PDF]
